# Relevance of the post-COVID syndrome within rehabilitation (PoCoRe): study protocol of a multi-centre study with different specialisations

**DOI:** 10.1186/s40359-022-00892-8

**Published:** 2022-07-29

**Authors:** Alexa Kupferschmitt, Thilo Hinterberger, Ida Montanari, Matthias Gasche, Christoph Hermann, Michael Jöbges, Stefan Kelm, Gerhard Sütfels, Andreas Wagner, Thomas H. Loew, Volker Köllner

**Affiliations:** 1 Research Group Psychosomatic Rehabilitation, Department of Psychosomatic Medicine, Charité Universitätsmedizin, Campus Benjamin Franklin, Hindenburgdamm 30, 12203 Berlin, Germany; 2 Rehabilitation Clinic Seehof, Department of Psychosomatic Medicine, Federal German Pension Agency, Lichterfelder Allee 55, 14513 Teltow, Germany; 3 Department of Psychosomatic Medicine, University Hospital Regensburg, Rilkestraße 39, 93049 Regensburg, Germany; 4 Centre for Pneumology and Psychosomatic Medicine, Ludwigstraße 68, 93093 Donaustauf, Germany; 5 Gelderland Clinic Geldern, Clemensstrasse 10, 47608 Geldern, Germany; 6 Schmieder Clinics Gailingen, Auf dem Berg 1, 78262 Gailingen, Germany; 7 Schmieder Clinics Constance, Eichhornstraße 68, 78464 Constance, Germany; 8 Westerwald Clinic Waldbreitbach, Buchenstraße 6, 56588 Waldbreitbach, Germany; 9 Todtmoos Rehabilitation Centre, Wehrawald Clinic, Federal German Pension Agency, Schwarzenbacher Straße 4, 79682 Todtmoos Todtmoos, Germany; 10 Alpcura Specialist Clinic Pfronten, Peter - Heel - Straße 29, 87459 Pfronten, Germany

**Keywords:** Post-COVID rehabilitation, Treatment effectiveness, Work ability, Fatigue, Post Exercise Malaise

## Abstract

**Background:**

In Patients suffering from post-COVID syndrome, in addition to physical limitations, cognitive limitations, fatigue, dyspnea as well as depression and anxiety disorders may also be present. Up to now (as of May 2022), approx. 514 million people worldwide have been infected with SARS-CoV-2, in Germany this affects approx. 25 million. In Germany, 2.5 million people could potentially be affected by post-COVID syndrome. Post-COVID is thus a highly relevant public health issue. So far, there is no specific causal therapy for the post-COVID syndrome, but with multimodal symptom-oriented rehabilitation, the course can be favourably influenced. However, there is no study yet that focuses on patients in different rehabilitation indications and compares the focal symptomatology and coping strategies as well as the patients' benefit per indication.

**Methods/design:**

As first objective, pulmonal, cardiac, neurological, cognitive or/and psychological functional impairments in rehabilitation patients after COVID-19 disease will be described. The second objective is the differentiated review of the specific rehabilitation measures, in the short term and in the longer term for the purpose of future prognoses and optimisation of therapeutic interventions. This prospective, non-randomised, controlled longitudinal study, plus multi-group comparisons will take place in seven rehabilitation clinics of different specialisations: cardiological rehab, pneumological rehab, neurological rehab, psychosomatic rehab. Within 12 months, 1000 cases across all participating centres will be included. Somatic and psychological testing will be conducted at three measurement points: Admission (t0), discharge (t1), 6-montas Catamnesis (t2). The patients receive the usual care according to the respective rehabilitation priorities, adapted to the special challenges of post-COVID symptoms. Patients of the post-COVID outpatient clinic without rehabilitation will be used as a control group.

**Discussion:**

This study will precisely assess the extent to which subclinical neurological or/and psychological impairments are present in post-COVID-19 rehabilitation and the results will help, developing, providing and evaluating appropriate treatment concepts. This may also have relevant implications for the improvement of physical ability and quality of life in post-COVID-19 patients and increase the probability of return to work.

*Trial registration* Z-2022-1749-8, registered 03. February 2022, https://studienanmeldung.zks-regensburg.de

## Background

In December 2019, the new coronavirus COVID-19 became known and led to a considerable number of worrying cases of illness being reported to the World Health Organisation [1] within a very short time (514 mio. cases worldwide until March 2022; 25 mio. cases in Germany). The WHO declared COVID-19 a pandemic on 11 March 2020. In Germany till March 2022, more than 25 million have been infected. So far, most of the initial infection has been mild to moderate, but late effects in the form of post-COVID syndrome have emerged in both severely affected and less dramatic cases.

The post-COVID syndrome is defined as a condition that usually occurs three months after presumed or confirmed SARS-CoV-2 infection and is characterised by symptoms that persist for at least two months and cannot be explained by any other diagnosis [2]. The most frequently complained symptoms are fatigue, shortness of breath and cognitive impairment, with frequently changing physical complaints and psychological symptoms also occurring in addition and the symptom picture affecting daily functioning in everyday life.

Studies indicate an incidence of post-COVID syndrome of 5–10% in mild to moderate courses of COVID-19 [3, 4], while it can reach 85% in hospitalised patients [5]. The sometimes very large variances in the prevalences found can be explained by the different study populations, symptoms and health problems recorded and follow-up periods. Another crucial aspect is whether the diagnosis of post-COVID syndrome is made on the basis of persistent symptoms associated with COVID-19 or whether the WHO criterion of functional impairment in various areas of life is taken into account. Population-based or sample-based studies, which also include milder courses of the disease, indicate a significantly lower incidence of long-term symptoms: 13.3% of the test-positive study participants had symptoms for longer than 28 days, 4.5% for longer than eight weeks and 2.3% for longer than 12 weeks [4].

Fatigue is the most common symptom, reported by 17.5–72% of post-COVID cases, followed by persistent dyspnoea (10–40%). Psychological problems, chest pain, and smell and taste disturbances can occur in up to 26%, 22% and 11% of patients, respectively [5]. Surprisingly, it has also been shown that people without SARS-CoV-2 infection also report persistent post-COVID-like symptoms [6], which illustrates the low specificity of the complaints and the need for interdisciplinary cooperation between specialists.

In a relevant proportion of those affected, this results in prolonged incapacity to work or, in the worst case, threatens their participation in working life [7]. In view of the SARS-CoV-2 infection figures, this represents a highly relevant problem both for those affected and for the health system [8]. Post-COVID is thus a highly relevant mental health and health care issue [9]. Psychomental limitations in turn interact with the somatic and have a considerable influence on the recovery process in terms of activity and participation and interaction with the social environment. So far, there is no specific causal therapy for the post-COVID syndrome, but with multimodal symptom-oriented interventions, the course can be favourably influenced [10, 11]. This results in the task, especially for rehabilitation, of developing, providing and evaluating appropriate treatment concepts. But with approximately 25 million COVID-19 cases in Germany, an estimate of 10% of those affected by post-COVID syndrome could be 2.5 mio people affected in Germany alone. With an estimate of 2% of those requiring rehabilitation, there would be 500.000 patients. This far exceeds for example the Germany-wide rehabilitation capacities of 150.000 psychosomatic treatment units.

The aim of this study would be to be able to precisely assess the extent to which subclinical somatic or/and psychological impairments are present in post-COVID-19 rehabilitation as soon as possible. It also appears to be of interest whether the rehabilitation success differs according to indication and setting (e.g. pneumology, neurology, etc.) in comparable outcome parameters (e.g. quality of life and functionality, depression and anxiety, somatic symptoms, cognitive performance level, etc.) and consequently whether statements about the (socio-medical) prognosis are possible.

This prospective, explorative, multicentre, mixed-methods study with clinical, standardised examination will include somatic and psychological testing at three measurement points: Admission (t0), discharge (t1), 6-month catamnesis (t2). The study is going to take place in Cardiological rehabilitation, Pneumological rehabilitation, neurological rehabilitation and Psychosomatic rehabilitation. This study is in line with the Stanford Hall consensus statement for post-COVID-19 rehabilitation [12]. In a structured and quantitative way the primary COVID-19, the current post-COVID symptomatology as well as the relevant history on quality of life and functionality will be recorded. Psychological stress, e.g. in the form of depression or anxiety, is assessed via questionnaires, and the cognitive performance parameters relevant to occupational medicine are assessed via an objective neuropsychological test battery. Fatigue and other somatic aspects of physical performance and the muscular/pneumological situation are recorded in the course of medical examinations. We outline that our study protocol is in line with the research agenda of post-COVID fatigue [13]. Furthermore, this study addresses the phenomenon of post-exercise malaise, which is postulated in the line to the post-COVID syndrome, but for which there is a lack of reliable data on whether or how often this phenomenon actually occurs. Apart from that, a training-focused approach is also recommended in this context. In the context of this study, we are therefore not only testing whether rehabilitation is effective, but also whether it is safe for patients with post-COVID syndrome.

The first objective is the description of neurological, cognitive or/and psychological functional impairments in rehabilitation patients after COVID-19 disease.

The second objective is the differentiated review of the specific rehabilitation measures, in the short term and in the longer term (6-month catamnesis) for the purpose of future prognoses and optimisation of therapeutic interventions / the therapeutic offer.

## Research questions

### Primary research question

- 1.1 Which neurological, internistic and psychosomatic symptoms are present in rehabilitation patients after a COVID-19 disease and how severe are they?
- 1.2 Do the three indications (neurology, pneumology, psychosomatics) differ with regard to the symptom severity of their rehabilitation patients?
- 1.3 How are the symptoms related to each other? Are there specific symptom clusters?

### Secondary research questions

- 2.1 To what extent do the respective symptoms (dyspnoea or reduced lung function; cognitive impairments; psychological complaints; functional impairments) change during rehabilitation?
- 2.1.1. Does the type of illness behaviour change during rehabilitation?
- 2.2 Are there differences in the different rehabilitative settings?

### Other research questions

- 3.1 To what extent does the different symptomatology (pneumological, neurological, psychosomatic) correlate with an unfavourable socio-medical evaluation.
- 3.2 Is the type of illness behaviour (avoidance-endurance) a predictor of symptomatology and socio-medical performance assessment?
- 3.2 Is there evidence of post-exercise malaise or other adverse events? Are there patients who deteriorate in the course of rehabilitation? (especially in the Six-Minute Walk Test)
- 3.4.1 Are there medical parameters that can be identified as risk parameters for a unfavourable socio-medical prognosis and a prolonged course of the condition (e.g. spiroergometry, lactate test)?

## Methods/design

### Participants, interventions, and study variables

#### Study setting and recruitment

**Fig. 1:**
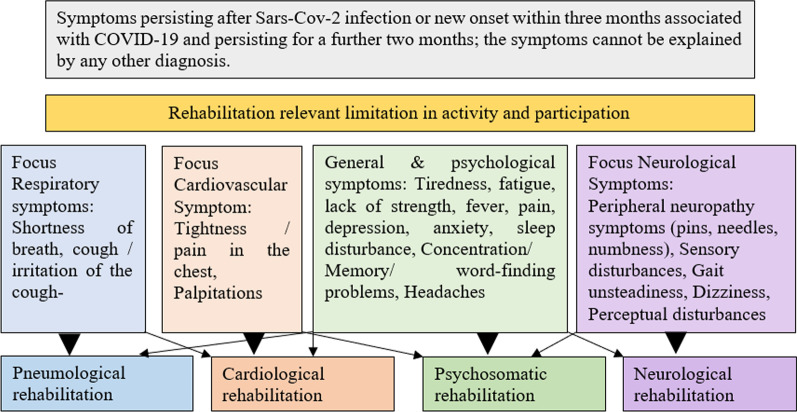
Allocation procedure

The envisaged study will take place in seven rehabilitation clinics of different specialisations: cardiological rehabilitation, pneumological rehabilitation, neurological rehabilitation, psychosomatic rehabilitation. All consecutively admitted rehabilitation patients who start rehabilitation as a result of COVID-19 and who meet the eligibility criteria as well as consent to participate in the study will be included. The previous course of infection of the affected persons is irrelevant for the inclusion in the study (e.g. mild symptoms to severe course with hospitalisation) (Fig. 1).

#### Eligibility criteria

- SARS-CoV-2-infection and following post-COVID syndrome: Complaints that are still present more than 12 weeks after the onset of SARS-CoV-2 infection and cannot be explained otherwise.
- As a consequence of the post-COVID syndrome, at the time of the start of rehabilitation, the presence of functional limitations that may threaten the ability to work.
- Written informed consent to participate in the study.
- Aged 18 years or above.
- Sufficient knowledge of the German language to participate in the study.

#### Interventions

Depending on the symptom focus, rehabilitation patients with post-COVID are treated in specialized rehabilitation clinics. The clinics and their treatment programs are listed below (Table 1). Since post-COVID syndrome is a new clinical picture with multi-organ involvement, attempts are currently being made to assign patients to already existing rehabilitation structures according to symptom focus. As can be seen, the specialisations diverge in the duration and frequency of the therapies, but uniformly cover all essential symptom areas of the post-COVID syndrome with corresponding offers (psychoeducation, exercise, psychotherapy, breathing and relaxation therapy, cognitive training).

**Table 1:** Interventions

Clinic	Psychoeducation	Exercise therapy /physical therapy	Relaxation	Psychotherapy		Respiratory therapy	Cognitive training	Creative therapy	Other
				Group	Single session				
*Rehabilitation Centre Todtmoos*									
Pneumological departement	1 × per rehabilitation/60 min *Post-COVID Pneumology* 1 × per rehabilitation/90 min *Post-COVID psychosomatic perspective* 1 × per rehabilitation/60 min *Introduction to exercise therapy*	3 × per week/30 min exercise therapy 5 × per week/30 min monitored ergometer training	3 × per week/30 min relaxation therapy 1 × per week/60 min QiGong (as needed)	2 × per rehabilitation/90 min	1 × per rehabilitation/45 min	4 × per week/30 min	As needed (Freshminder)	Ergotherapy (as needed) Art therapy (as needed)	1 × per week/60 min pacing-group Olfactory training (as needed) Nutrition advice (as needed) Social service (as needed)
Psychosomatic Department		1 × per week/90 min exercise therapy 4 × per week/30 min monitored ergometer training	2 × per week/30 min relaxation therapy	2 × per week/90 min 1 × per week/60 min	1 × per week/30 min	2 × per week/30 min		1 × per week/90 min Ergotherapy 1 × per week/90 min Art therapy	
Psycho-pneumological departement		2 × per week/60 min exercise therapy 4 × per week/30 min monitored ergometer training	2 × per week/30 min relaxation therapy	3 × per week/90 min	1 × per week/60 min	5 × per week/30 min		1 × per week/90 min Ergotherapy	
*Alpcura Clinic*									
Pneumological rehabilitation All Patients	1 × per rehabilitation/60 min *Post-COVID* 1 × per rehabilitation/30 min *social law / medicin* 3 × per rehabilitation/60 min *diet* 1 × per rehabilitation/30 min *Hygiene training*	2 × per week/30 min monitored ergometer training 2 × per week/30 min strenght training 2 × per week/30 min Vibration platform training 3 × per rehabilitation 45 min back school 1 × per week 45 min Balance training (as needed)	2 × per week/30 min Relaxation therapy 1 × per week/20 min Hydrojet massage 1 × per week/25 min infrared treatment	1 × per week/60 min (as needed)	1 × per week/30 min (as needed)	2 × per week/45 min	3 × per rehabilitation/ 60 min	1 × per rehabilitation/ 60 min Daily life training (as needed) 3 × per rehabilitation/ 60 min technique/skills training (as needed)	30 min Water stepping (as needed) Social service (as needed) 1 × per week/ 45 min conversation group „think positive “ (as needed)
Low capability patients		3 × per rehabilitation/ 45 min. Stair climbing under physiother. Guidance and pulsoxymetric monitoring 1 × per week functional training						1 × per rehabilitation (or more) Ergotherapy	
More powerfull patients		1 × per rehabilitation/45 min Facia training 1 × per week/45 min spinal gymbastics 1 × per week/45 min cardiovascular training 1 × per week/ min Nordic Walking	2 × per week/45 min Relaxation therapy 3 × per week/30 min QiGong					None	
*Rehabilitation Clinic Seehof, Teltow *									
Psycho-cardiolocical rehabilitation	1 × per rehabilitation/60 min *Post-COVID* 1 × per rehabilitation/30 min *Introduction to exercise therapy* Mulltiple times per rehabilitation/60 min *psychosomatic or cardiological topics* 1 × per rehabilitation/45 min *Introduction cognitive training*	2 × per week/30 min exercise therapy „COfit “ 2 × per week/30 min monitored ergometer training 2 × per week/45 min Nordic Walking (as needed) 2 × per week 30 min aqua fitness (as needed)	2 × per week/45 min Relaxation therapy 1 × per week/60 min QiGong Hydrojet massage (as needed)	2 × per week/90 min	2 × per week/30 min or 1 × per week/60 min	2 × per week/30 min	2 × per week/60 min Group 3 × rehabilitation/30 min Single session Accompanying training tasks	1 × per week/ 90 min Ergotherapy Art therapy (as needed) Therapeutic dance (as needed)	Nutrition advice (as needed) Social service (as needed) 2 × per week/ 30 min Mindfulness group Aroma therapy (as needed)
*Gelderland Clinic*									
Psychosomatic rehabilitation	1 × per rehabilitation/60 min *Post-COVID and Naturotherapy* 2 × per rehabilitation/60 min *Naturotherapy for stress at work* 1 × per rehabilitation/60 min *Introduction cognitive training* 1 × per rehabilitation/30 min *Introduction cognitive training Freshminder*	1 × per week/45 min monitored strenght training 1 × per week/45 min monitored coordination training or 1 × per week/30 min aqua fitness 1 × per week/60 min Walking	1 × per week/45 min QiGong or Yoga 1 × per week/45 min meditative moving	2 × per week/ 75 min 1 × per week/ 60 min	1 × per week/30 min	2 × per week/30 min Group 2 × per rehabilitation/ 30 min single session	2 × per week/30 min Group	1 × per week/45 min meditative dancing or 1 × per week/45 min meditative forest bathing 1 × per week/30 min dew stepping Ergotherapy (as needed)	Nutrition advice (as needed) Social service (as needed) 1 × per week/ 30 min Mindfulness group Aroma therapy (as needed)
*Schmieder Clinics*									
Neurological rehabilitation *Note.* Patients have approx. 19–21 therapy sessions per week in an individual therapy plan	1 × per rehabilitation *Post-COVID* Multiple times per rehab, different topics like *smoking cessation, dietetics, overweight, muscular deficits*	After performance level assessment: Stamina training Strength training Vibration platform training	As needed (PMR)	As needed	As needed	Breathing therapy group Guided self-exercise training with breathing therapy device Oxygen therapy (as needed)	Assessment and training as needed	Daily life training (as needed) motor-functional training (as needed)	Olfactory training (as needed) Nutrition advice (as needed) Social service (as needed)
*Clinic Westerwald*									
Neurological rehabilitation *Note.* Patients have a very individual therapy plan	1 × per rehabilitation *Post-COVID* Multiple times per rehab, different topics like *fatigue, headache, dietetics,* *Smoking cessation*	After performance level assessment: stamina training strength training Vibration platform training	As needed (PMR, autogenic training)	none	1 × per week/ 60 min (as needed)	As needed	Assessment and training (CogniPlus) as needed	Compensatory and memory strategies (as needed)	Olfactory training (as needed), including Kaiteki maneuver with local application of vitamin A Speech therapy (as needed) Social service (as needed

The rehabilitation concepts of the neurological clinics are so individual that it is not possible to make any useful and schematic statements about therapy times in advance

### Control group (post-COVID outpatient clinic Regensburg)

This is a matched control group or comparative data from Regensburg Post-COVID Outpatient Clinic. The patients in the Regensburg post-COVID outpatient clinic meet the same inclusion criteria as the study cohorts of the rehabilitation clinics and receive the same diagnostic examinations. Due to the ambulance setting, which focusses only on the diagnostic process, there is no intervention.

#### Study variables

The study-related examinations/tests are carried out by a psychological specialist upon admission to rehabilitation and discharge. If spiroergometry is carried out on the patients, this is done by the specialist staff and the findings are added to the study documentation by the local study assistant. At discharge, a treatment recommendation is made according to the findings available at that time, e.g. short versus long neuro-rehabilitation (in addition to the somatic, psychosomatic and/or integrated rehabilitation). Within the framework of the catamnesis, all further medical measures that were used during the observation period are recorded retrospectively.

#### Measures/outcomes

Table 2 provides an overview of the assessments used.

**Table 2:** Overview of the assesments used

Instruments	Domains covered	Items
*Psychological assessment*		
Regensburg COVID documentation (ReCoRD)	Primary COVID-19 symptoms, current post-COVID symptoms (25 frequently reported complaints of different organ systems); relevant medical history	25
Work ability index (WAI) Ilmarinen [14]	Demands of the job, health status, resources of the worker	7
Personal health questionnaire (PHQ) Spitzer et al. [15]	Depressive, anxiety,somatoform disorders, psychosocial functionality, stressors, critical life events	78
Dissability assessment (WHO-DAS 2.0) Üstün et al. [16]	Cognition (understanding & communicating), Mobility (moving & getting around), Self-care (hygiene, dressing, eating & staying alone), Getting along (interacting with other people), Life activities (domestic responsibilities, leisure, work & school) and Participation (joining in community activities)	36
Life skills (LK-18). Hinterberger, Walter and Galuska [17]	Well-being, self-regulation, commitment, sense of purpose, self-efficacy and social contacts	18
Fatigue scale for motor functioning and cognition (FSMC) Penner et al. [18]	Subjectively experienced fatigue symptoms, graduation of cognitive and motor fatigue	20
Avoidance-endurance questionnaire (AEQ). Hasenbring et al. [19] (alternatively: Avoidance-Endurance Questionnaire, short Version (AE-FS).)	Fear-avoidance responses and endurance-related responses (cheerful-suppressive way and a distress-endurance way)	49
*Neurological assessment*		
Montreal cognitive assessment (MoCA). Nasreddine et al. [20]	Various cognitive abilities such as memory, language, contextual thinking, attention and concentration, behaviour, arithmetic, temporal and spatial orientation and the ability to recognise complex shapes and patterns	30
Test battery for attention (TAP) Scherwath et al. [21]	Intensity of attention, Executive functions, control of the focus of attention, Attentional selectivity, focused attention, visuo-spatial attention	45 min
*Somatic assessment*		
Six-Minute walk test (6MWT) Enright [22]	Assess and control cardiovascular and pulmonary performance below the anaerobic threshold	
Spiroergometry Kroidl et al. [23]	Function of the heart, circulation, respiration and muscle metabolism in a relaxed state and under increasing physical stress up to maximum load	
Pulmonary function test [24, 25]	Functional and performance capacity of the lungs and bronchial tubes; monitoring the course and therapeutic success of lung diseases	
Blood gas analysis and laboratory (blood test) Haber [26], Boemke et al. [27]	Blood test that measures how much carbon dioxide and oxygen are in the blood; conclusions can be drawn about the health of the heart and lungs	
Caridac echo Picard and Weiner [28] Carmeli et al. [29]	Information about the structure of the heart (e.g. size of the heart chambers, function of the heart valves, thickness of the heart muscle)	
24-h ECG Mehraeen et al. [30], Ståhlberg et al. [31]	Information about how the heart functions under everyday conditions	
*Post-exercise malaise/adverse events monitoring*		
Structured recording of adverse event/fatigue	Fatigue level, post-exercise malaise / adverse events	

#### Psychological assessment

##### The Regensburg COVID documentation (ReCoRD)

The ReCoRD is a self-report questionnaire and is used for the structured and quantitative assessment of the primary COVID-19 symptoms, the current post-COVID symptoms and the relevant medical history. A total of 25 frequently reported complaints of different organ systems in the context of post-COVID are recorded (e.g. cough, shortness of breath, palpitations, headaches, concentration disorders, depression, sleep disturbance, odour/taste disorders, neuropathy). The presence is recorded dichotomously. Furthermore, it is asked to what extent the symptoms were already present before, how long they have existed since the COVID-19 illness, as well as the frequency and current intensity of the complaints.

##### Work ability index (WAI)

The WAI is a well-established instrument used in clinical occupational medicine and research to assess work ability during health examinations and workplace surveys. The index is determined based on the answers to a series of questions assigned to the seven WAI dimensions, which take into account the demands of the job, the health status and the resources of the worker (Current work ability compared with the lifetime best; Work ability in relation to the demands of the job; Number of current diseases diagnosed by a physician; Estimated work impairment due to diseases; Sick leave during the past year (12 months); Own prognosis of work ability two years from now; Mental resources) [14]. The WAI has sufficient psychometric properties [14, 32, 33].

##### Personal health questionnaire (PHQ)

The PHQ is an internationally established questionnaire screening for the systematic assessment of mental disorders. The complete version consists of a total of 78 items, which are divided into 16 sections. The items are assigned to different response categories (dichtotome yes–no questions; three- to four-level ordinal scales). The structure of the PHQ is modular and thus enables targeted screening of depressive, anxiety or somatoform disorders, as well as eating disorders and alcohol abuse. In addition, questions on psychosocial functionality, stressors, critical life events and a gynaecological history (menstruation, pregnancy, birth) can be recorded, as well as global functional limitations due to psychological symptoms [15]. In this study, all modules except eating disorders, alcohol abuse and the gynaecological history are used.

##### Disability assessment (WHO-DAS 2.0)

The WHODAS 2.0 is a self-assessment instrument for health and disability, which can be used across all diseases, including mental, neurological and addictive disorders. The instrument covers six domains of functioning, including Cognition (understanding & communicating), Mobility (moving & getting around), Self-care (hygiene, dressing, eating & staying alone), Getting along (interacting with other people), Life activities (domestic responsibilities, leisure, work & school) and Participation (joining in community activities). The domains are directly linked at the level of the concepts to the International Classification of Functioning, Disability and Health (ICF). In this study we use the more detailed 36-item version, which can be self-administered in paper–pencil format. The items are assigned to a five-level ordinal scale (none–0, mild–1, moderate–2, severe–3, extreme–4); the summary score indicates the extent of impairment [16, 34].

##### Life skills (LK-18)

The life skills questionnaire LK-18 is a self-assessment instrument developed on the basis of psychosomatic inpatients, which covers the areas of well-being, self-regulation, commitment, sense of purpose, self-efficacy and social contacts by means of 18 items, which can be divided into 6 factors with 3 items each. The items are rated on a 5-point scale. The recorded life skills represent abilities from which a salutogenic lifestyle can result, which in turn can serve as a basis and important indicator for the therapeutic process [17].

##### Fatigue scale for motor functioning and cognition (FSMC)

The FSMC is an assessment which grasps subjectively experienced fatigue symptoms and which provides differential quantification and graduation of cognitive and motor fatigue. The FSMC was tested against several external criteria (e.g. cognition, motivation, personality and other fatigue scales) and provides satisfactory results with regard to the test quality criteria. Twenty items (10 for motor fatigue, 10 for cognitive fatigue) are rated using a five-point scale (strongly disagree to strongly agree) [18].

##### Avoidance-endurance questionnaire (AEQ)

The Avoidance-Endurance Questionnaire [19] uses 49 items to assess behaviours that may contribute to chronicity of symptoms. A distinction can be made between two processing modes: fear-avoidance responses and endurance-related responses (cheerful-suppressive way and a distress-endurance way). The cheerful-suppressive way involves focused forms of cognitive distraction from symptoms, maintaining a positive mood despite suffering, and continuing daily activities even in the presence of intense distress (eustress-endurance responses). The other way (distress-endurance responses) is characterised by unspecific suppression of symptoms and thoughts about the symptoms, heightened irritable and depressed mood, but still strong endurance during and despite the experience of not only mild but also severe distress. The scales referring to fear avoidance responses are anxiety/depression, catastrophizing, help-/hopelessness, avoidance of social activities, avoidance of physical activities. The scales referring to endurance-related responses comprise positive mood, thought suppression, pain persistence behavior and humor/distraction. Items are rated on a seven-point scale (never to always). The AE-FS [35] is a fast screening based on the AEQ and contains 9 items. Both instruments have satisfactory psychometric properties.

#### Neurological assessment

##### Montreal cognitive assessment (MoCA)

The MoCa test is performed by healthcare professionals to detect early stages of dementia and mild cognitive impairment and to distinguish them from “normal” senile forgetfulness. The MoCa test is significantly more accurate in detecting mild forms of dementia with only slightly impaired abilities than other well-established procedures such as the MMST, which is often used in dementia diagnostics. The test, which lasts about 10 min, contains 30 questions that test various cognitive abilities such as memory, language, contextual thinking, attention and concentration, behaviour, arithmetic, temporal and spatial orientation and the ability to recognise complex shapes and patterns. Scores of 26 to 30 points are considered unremarkable, no limitations, 6 to 25 points indicate at least mild cognitive impairment, and 0 to 5 points are interpreted as extreme mental impairment [20].

##### TAP-test

The Test Battery for Attention (TAP) is a software package and offers a collection of different test procedures with which the various sub-aspects of attention can be recorded in a differentiated manner and also cover related aspects of visual perception. Subtests are Alertness, working memory, sustained attention, flexibility, divided attention and go/nogo paradigm. The test takes about 45 min to complete [21] (Table 3).

**Table 3:** Overview of the TAP subtests used

Subtest	Cognitive domain	Information about
Alertness	Intensity of attention	Basal reactivity, general processing speed, reaction stability, distinction tonic and phasic alertness
Working memory	Executive functions, control of the focus of attention	Ability to continuously update the content of working memory
Sustained attention	Intensity of attention	Longer-term maintenance of attention with high target stimulus density
Divided attention	Attentional selectivity, focused attention, visuo-spatial attention	Ability to focus attention on two tasks simultaneously
Alertness	Intensity of attention	See above

#### Somatic assessment

##### Six-minute walk test (6MWT)

The 6MWT is used to assess and control cardiovascular and pulmonary performance below the anaerobic threshold. In the test, the patient has to walk for 6 minutes over an incline-free circuit or a walkway of at least 30 meters in length. The goal for the patient is to cover as much distance as possible in the given time window. The distance is measured in metres [22]. In addition, just before the start of the 6MWT in a standing position and at the end of the 6 minutes, respiratory distress is determined using the Borg CR10 scale [36].

##### Spiroergometry

The spiroergometric examination assesses the function of the heart, circulation, respiration and muscle metabolism in a relaxed state and under increasing physical stress up to maximum load. By simultaneously measuring the oxygen and carbon dioxide concentration in the inhaled and exhaled air, oxygen consumption (VO2) and carbon dioxide production (VCO2) can be determined [23].

##### Pulmonary function test

The pulmonary function test assesses the functional and performance capacity of the lungs and bronchial tubes. It can be used for monitoring the course and therapeutic success of lung diseases [25] and other respiratory tracts [37].

##### Blood gas analysis and laboratory (blood test)

Blood gas analysis can be used to measure how much carbon dioxide and oxygen is in the blood, and conclusions can be drawn about the health of the heart and lungs. The blood count gives an overview of the cellular components contained in the blood and allows an assessment of whether diseases are present that are initially asymptomatic [26, 27].

##### Cardiac echography and 24-h ECG

Echocardiography is one of the most important routine heart examinations. The ultrasound examination provides information about the structure of the heart and makes it possible, for example, to assess the size of the heart chambers, the function of the heart valves or the thickness of the heart muscle [28, 29]. The 24-h ECG records the electrical heart actions over mostly 24-h and thus enables the diagnosis of recurrent disorders, especially cardiac arrhythmias [30, 31].

#### Post-exercise malaise/adverse events monitoring

##### Structured recording of adverse events/fatigue

In the psychotherapy group, the fatigue level and adverse events that result in post-exercise malaise are recorded and logged in a structured manner with the addition of behavioural analysis.

### Data management and analysis

#### Data management

**Fig. 2:**
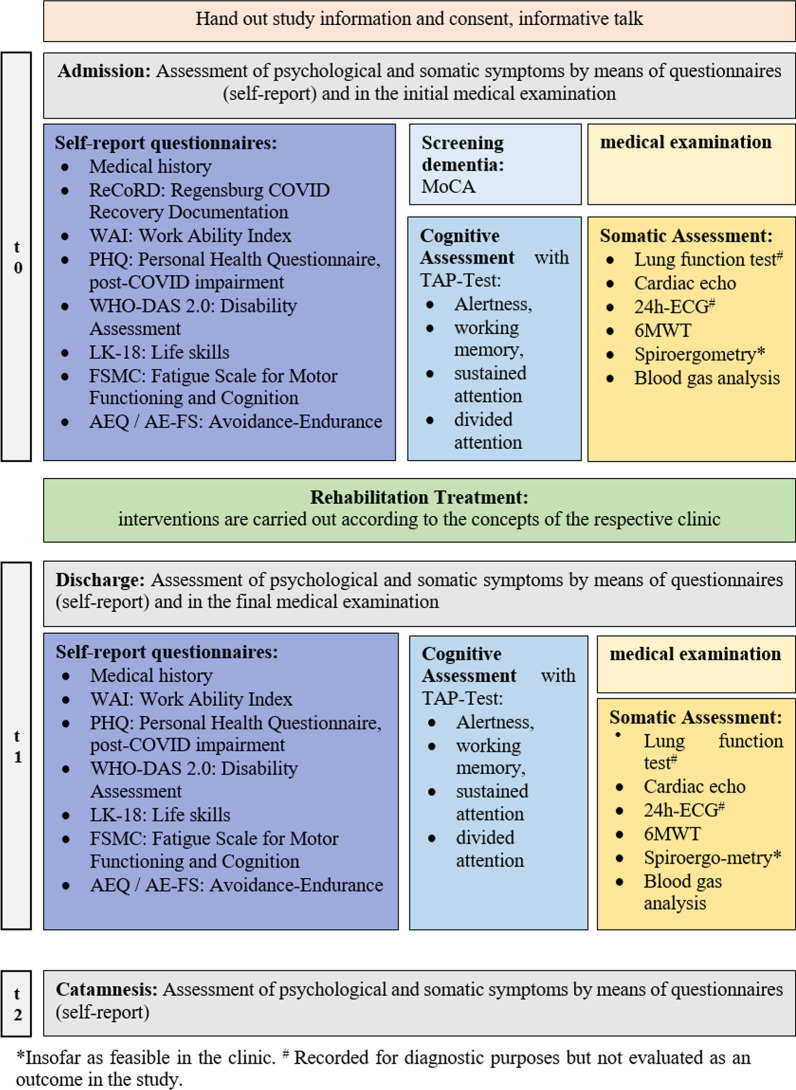
Study procedure

Outcomes and other measures will be assessed with patient questionnaires and cognitive assessment, with medical examination and somatic assessment (see Fig. 2) or will be extracted from the rehabilitation discharge letters (e.g. primary and demographic data; socio-medical parameters e.g. employment, current sickness absence, sick leave duration in weeks, ability to work on admission and discharge). For participants who do not complete follow-up questionnaires, missing data will be imputed. If participants withdraw their participation, the collected data will be deleted. The patient data are stored anonymously in a electronic study-data file with a patient cipher, whereby the paper–pencil data are entered manually. Paper-pencil questionnaires will be stored in locked filing cabinets and electronic data will be stored on secured servers. To assure a safe and secure environment for data acquired, data transmission is encrypted with secure socket layer (SSL) technology.

#### Statistical methods

Statistical analyses will be conducted using R version 4.0.5 [38], the package lavaan [39], Matlab [40], and/or SPSS 21 or 28 [41] for Windows.

#### Descriptive

Descriptive and explorative analyses will be performed concerning demographic, disease-specific (diagnosis; neurological, cognitive or/and psychological functional impairments) and other factors (e.g. work related factors like ability to work on admission, duration of sick-leave).

#### Treatment effect

Differences in the change scores between the intervention groups (reha-clinics) and the control group (post-COVID ambulance) on the primary (self-report questionnaires, screening dementia, cognitive assessment, somatic assessment) and secondary outcomes (socio medical parameters) will be analysed with multilevel (mixed-model) linear regression analyses taking all three measurements into account (admission, discharge, catamnesis). We will use a model with a random intercept and random slopes will be tested, to see which model fits best. *P* values < 0.05 are considered statistically significant. Differences in the change scores between the intervention groups and the control group on the primary, secondary and other study parameters will be analysed with multilevel (mixed-model) linear regression analyses, taking all three measurements into account. We will perform subgroup analyses for severity, duration and number of the symptoms and comorbidity to explore which patients are most likely to benefit from the intervention.

## Discussion

Post-COVID syndrome affects a significant proportion of the working population worldwide and there is as yet no specific causal therapy. Recovery also tends to be a long process, making it difficult to re-enter social and working life. However, data to date show that the course can be favourably influenced with multimodal symptom-oriented interventions [10, 11]. This leads to the task, especially for rehabilitation, of developing, providing and evaluating appropriate treatment concepts. The questions arise as to how suitable possibilities for easily accessible diagnosis and therapy of post-COVID can be created for post-COVID patients, what connections exist between post-COVID symptoms and other health problems that may also be present, and to what extent psychosomatic factors play a role in post-COVID symptoms. In particular, chronic fatigue, depression and anxiety, as well as cognitive problems, often occur and will be investigated in more detail in this multi-centre study with several rehabilitation clinics for different indications. As treatment programs for post-COVID are emerging, it seems particularly relevant to examine the impact of the various interventions currently offered to post-COVID patients on post-COVID symptoms. It needs to be clarified whether in some cases additional neurological, psychosomatic or internal rehabilitation treatment in a suitable clinic is required and, if so, how frequent such cases are. In order to ensure the best possible care, an answer to the question of whether indications of particularly suitable therapy offers can be found on the basis of the patients’ current findings and their medical history would be important.

As post-COVID symptoms and especially post-COVID fatigue often appear to have profound effects on daily functioning, including work ability and quality of life, significant economic impacts are also expected through increased sick leave and use of health care, as is already the case for patients with infectious diseases other than COVID-19 [42, 43]. Given the global scale of this pandemic and the large group of potential patients, special attention must be paid to how society as a whole will be affected.

### Strength and limitations

Following the recommendation of the research agenda for post-COVID fatigue [13], this study uses largely internationally established and previously validated instruments to measure fatigue and other post-COVID symptoms. The strengths of this study include the large sample size and the nationwide distribution of the cooperating rehabilitation centres, which thus also reflects regional differences and characteristics of the various rehabilitation indications. The inclusion of different therapy focuses per indication with different intensity and duration of treatment is on the one hand a weakness of the study, as no uniformly defined intervention can be depicted, but on the other hand it reflects the reality of healthcare. This improves the external validity of our study results. The resulting comparison between the different care structures also enables care research to be conducted within this study. We assume that within the framework of this observational study, statements can be made reliably about which and how much therapy the respective patient groups need. With this approach, the treatment of the control group resembles usual care and offers the possibility to make statements about whether pure post-COVID diagnostics might be sufficient for certain patient groups.

However, external validity outside Germany will be limited due to differences in health care systems. A further relevant weakness is that there is no RCT design, but this is difficult for ethical and legal reasons, first because in Germany everyone has a legal right to rehabilitation (entitlement to rehabilitation) and second because rehabilitation is relevant to clarify the socio-medical situation.

## Conclusion

This protocol describes a multi-centre, non-randomised, controlled longitudinal study, plus multi-group comparisons, which will take place in seven rehabilitation clinics of different specialisations. By providing a detailed diagnosis of post-COVID symptoms and an indicative treatment tailored to the special needs of post-COVID patients, this study aims to determine the respective needs of symptomatically different affected persons for treatment options, to answer the question of which patient groups need which doses of treatment and which interventions, and at which points modifications should be made in the treatment concepts in order to ensure an optimal therapy. Referring to the high prevalence of post-COVID syndrome [2], their large burden of disease [5, 10] and economic impact on labour markets and social security systems [7, 8], a positive evaluation of the intervention, detection of possible adverse events and subsequent implementation into practice could be of large mental and public health and economic relevance.

## Data Availability

Since the data that will be used in the targeted study in the future will be data on insured persons of the German Pension Insurance and strict data protection exists, these data are not publicly accessible, but can be made available by the corresponding author in a form adjusted for personal data upon justified request.

## Abbreviations

6MWT: Six-minute walk test
AEQ/AE-FS: Avoidance endurance questionnaire
ECG: Echocardiography
FSMC: Fatigue scale for motor functioning and cognition
LK-18: Life skills
MoCA: Montreal cognitive assessment
PHQ: Personal health questionnaire
PoCoRe: Post COVID rehabilitation
ReCoRD: Regensburger COVID recovery documentation
TAP: Test battery for attention
WAI: Work ability index
WHO-DAS 2.0: Disability assessment
